# Inverse-phase composite zone plate providing deeper focus than the normal diffraction-limited depth of X-ray microbeams

**DOI:** 10.1107/S1600577518016703

**Published:** 2019-01-01

**Authors:** Yasushi Kagoshima, Yuki Takayama

**Affiliations:** a Graduate School of Material Science, University of Hyogo, 3-2-1 Kouto, Kamigori, Ako, Hyogo 678-1297, Japan

**Keywords:** zone plate, diffraction limit, depth of focus, spatial resolution, microbeam

## Abstract

A novel type of zone plate (ZP), termed an inverse-phase composite ZP, is proposed to gain a deeper focus than the standard diffraction-limited depth of focus, with little reduction in spatial resolution. The structure is a combination of an inner ZP functioning as a conventional phase ZP and an outer ZP functioning with third-order diffraction with opposite phase to the inner ZP. Two-dimensional complex amplitude distributions neighboring the focal point were calculated using a wave-optical approach of diffraction integration with a monochromatic plane-wave illumination, where one dimension is the radial direction and the other dimension is the optical-axis direction. The depth of focus and the spatial resolution were examined as the main focusing properties. Two characteristic promising cases regarding the depth of focus were found: a pit-intensity focus with the deepest depth of focus, and a flat-intensity focus with deeper depth of focus than usual ZPs. It was found that twice the depth of focus could be expected with little reduction in the spatial resolution for 10 keV X-ray energy, tantalum zone material, 84 nm minimum fabrication zone width, and zone thickness of 2.645 µm. It was also found that the depth of focus and the spatial resolution were almost unchanged in the photon energy range from 8 to 12 keV. The inverse-phase composite ZP has high potential for use in analysis of practical thick samples in X-ray microbeam applications.

## Abstract 2

A novel type of zone plate, *i.e.* an inverse-phase composite zone plate, is proposed and examined with the aim of achieving deeper focus with little reduction in spatial resolution.

## 1. Introduction   

Fresnel zone plates (ZPs) are major optical elements in X-ray microscopes. There are several derivations such as sputtered-sliced ZPs (Rudolph *et al.*, 1982 ▸; Koyama *et al.*, 2012 ▸), multilayer Laue lenses (Maser *et al.*, 2004 ▸; Koyama *et al.*, 2008 ▸) and total reflection ZPs (Takano *et al.*, 2010 ▸). There are other types of optical elements such as grazing-incidence total reflection mirrors of Kirkpatrick–Baez mirrors (Mimura *et al.*, 2007 ▸) and Wolter type I mirrors (Aoki *et al.*, 1992 ▸), compound refractive lenses (Schroer *et al.*, 2005 ▸) and Bragg–Fresnel lenses (Erko *et al.*, 1994 ▸). The development of these optical elements has made sub-100 nm-spatial resolution easily available in X-ray microscopes. As the spatial resolution becomes higher, the demand for localized analysis of practical samples is rapidly increasing. However, according to the physical principle of the diffraction limit, the higher the spatial resolution (the smaller the focused beam size), the shallower the depth of focus in a focusing optical system. Optical elements have to date been unable to evade the diffraction limit. The diffraction limit in optics, which corresponds to the uncertainty principle in quantum mechanics, is usually expressed as being that the product of the spatial resolution (Δ_res_) and the numerical aperture (NA) cannot be smaller than the wavelength, namely, Δ_res_ × NA ≥ λ. Because the depth of focus (DoF) is also determined by NA such that DoF ∝ 1/NA^2^, improved spatial resolution and deeper DoF are incompatible. This can be interpreted as an alternative expression of the diffraction limit. This limit restricts the thickness of samples to be analyzed and is an impediment to high-spatial-resolution micro-analysis of practical samples using X-ray microscopes. In particular, the DoF is a critical parameter for X-ray micro-CT (computed tomography) with X-ray microscopes. The DoF of a ZP was well studied both with simulations and experiments aiming at high-spatial-resolution X-ray micro-CT (Wang *et al.*, 2000 ▸).

A novel type of ZP has been proposed, named an inverse-phase composite ZP (IP-CZP), in order to relax this limit, with an initial solution that facilitates a deeper DoF with little reduction of the spatial resolution (Kagoshima & Takayama, 2018 ▸). This paper reports on the focusing properties of the IP-CZP from the point of view of both DoF and spatial resolution.

It should be noted here that another way to produce a blurred focal spot with an extended DoF has been recently reported using multiple zone plate stacking with misalignment along the optical axis (Li & Jacobsen, 2018 ▸).

## 2. Inverse-phase composite ZP   

### 2.1. Principle and strategy to make DoF deeper   

**Figure 1:**
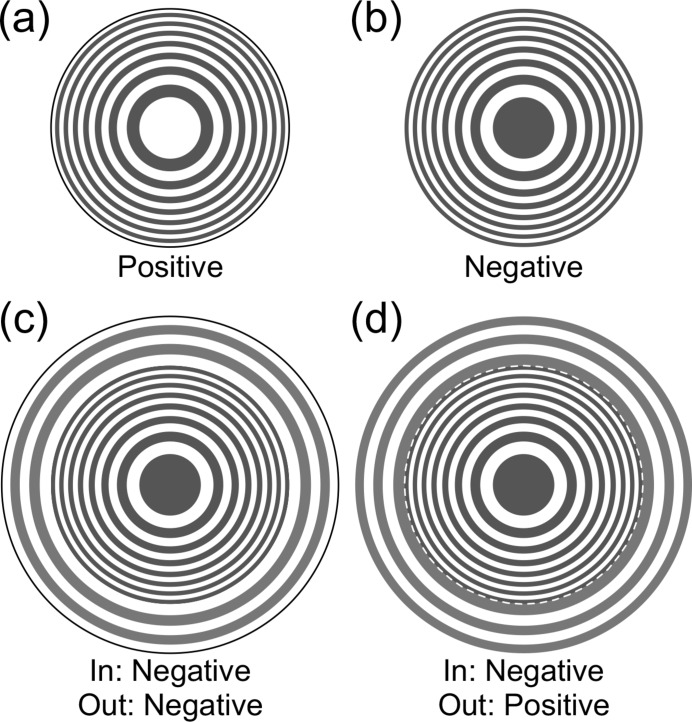
(*a*) Positive ZP, (*b*) negative ZP, (*c*) composite ZP and (*d*) inverse-phase composite ZP (IP-CZP). The gray color of the zones indicates that ZPs function as a phase zone plate. The white dotted circle in (*d*) is the boundary of the iZP and oZP.

There are two types of ZPs, positive and negative, as shown in Figs. 1(*a*) and 1(*b*) ▸. Both ZPs function as thin lenses. They have identical focal intensity distributions in the focal plane and along the optical axis, that determine Δ_res_ and DoF, respectively. On the other hand, the phase of the complex amplitude of focusing waves is opposite to each other. A composite ZP was proposed, and its optical properties were well studied (Michette, 1986 ▸). If the inner ZP with a first-order focal length of *f* is surrounded by the outer ZP with a third-order focal length also of *f*, the effective NA becomes larger as shown in Fig. 1(*c*) ▸. This leads to higher spatial resolution (smaller focused beam size) and an increase of the focused intensity.

In order to deepen the DoF without reducing the spatial resolution, the focal intensity distribution along the optical axis should be broadened, whereas the focusing beam size in the focal plane should be as unchanged as possible. Our strategy to accomplish this is as follows. We previously proposed that the phase of the inner ZP (iZP) and outer ZP (oZP) are inversely composed as shown in Fig. 1(*d*) ▸ (Kagoshima & Takayama, 2018 ▸). Because the aperture of the oZP is annular, the focused beam size produced only by the oZP becomes narrower than that of a circular aperture with the same NA. As the effective number of zones of the oZP becomes smaller, the monochromaticity requirement for the oZP becomes looser, which leads to the broadening in the focal intensity distribution of the oZP along the optical axis. The diffraction efficiency of ZPs depends on the zones’ material and thickness. If opaque zones act as phase material, ZPs work as phase ZPs (Kirz, 1974 ▸). By tuning the thicknesses of the iZP and oZP independently, the DoF and Δ_res_ of the IP-CZP can be controlled because the complex amplitude of the IP-CZP is the coherent sum of each complex amplitude of the iZP and oZP. Therefore, if the parameters of the IP-CZP are appropriately chosen, the DoF could be deepened with little reduction of the spatial resolution.

### 2.2. Definitions of design parameters   

**Figure 2:**
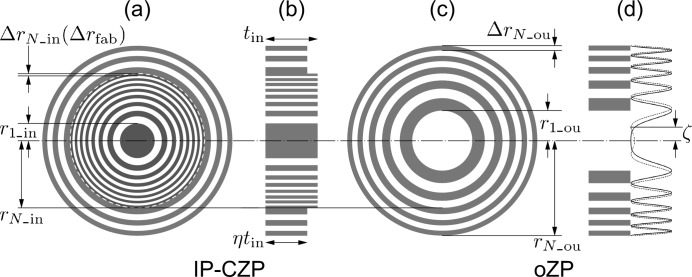
Definition of the main design parameters of the IP-CZP. (*a*) IP-CZP front view and (*b*) section; (*c*) outer ZP front view and (*d*) section.

Definition of the main variable design parameters of the IP-CZP are illustrated in Fig. 2 ▸. Those of the iZP are the first zone radius, *r*_1_in_, outer radius, *r*_*N*_in_, outermost zone width, Δ*r*_*N*_in_, which is supposed to be equal to the minimum fabrication width Δ*r*_fab_, and zone thickness, *t*_in_. Those of the oZP are the first zone radius, *r*_1_ou_, outer radius, *r*_*N*_ou_, outermost zone width, Δ*r*_*N*_ou_, and zone thickness, η*t*_in_, where η is the thickness ratio. In this paper, it is assumed that η ≤ 1. *r*_*N*_ou_ is identical to an outer radius of IP-CZP and represented by an annular parameter ∊ as *r*_*N*_ou_ = *r*_*N*_in_/∊. The focal length of the first-order diffraction of iZP, *f*_1_in_, is given by 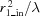. Since the focal length of the third-order diffraction of the oZP, *f*_3_ou_, is equal to *f*_1_in_, *r*_1_ou_ is 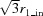. The local diffraction efficiency is determined by the refractive index of the zone material, *n* = 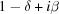, and the zone thickness (Kirz, 1974 ▸). The initial phase of oZP, ζ, should also be able to be tuned for the optimization. When ζ = π and η = 1, the IP-CZP is identical to a CZP. The effective number of zones of the oZP, *N*_ou_eff_, is 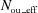 = 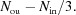


### 2.3. Spatial resolution (Δ_res_) and DoF   

The two main focusing properties of optical elements are the spatial resolution (Δ_res_) and the DoF. The former can be evaluated from the radial intensity distribution in the focal plane perpendicular to the optical axis. The latter can be evaluated from the intensity distribution along the optical axis neighboring the focal point. Both are defined by the numerical aperture (NA) as follows,
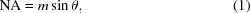


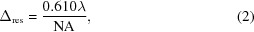


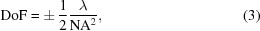
where *m* is the refractive index of the object space and θ is the objective angular semi-aperture. Equation (2) is well known as the Rayleigh criterion and usually understood to express the diffraction limit in microscopes. Δ_res_ is the same as the radius of the first null of the Airy pattern. DoF corresponds to a range within which the on-optical-axis intensity decreases by 20% from the peak intensity (Born & Wolf, 1986 ▸). Therefore, the smaller the Δ_res_, the shallower the DoF as given by
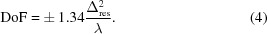
As described above, equation (4) is another expression of the diffraction limit for a circular aperture.

For a usual ZP, Δ_res_ and DoF can be given by the outermost (narrowest fabricated) zone width, Δ*r*_*N*_, as follows (Attwood, 1999 ▸),
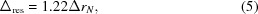


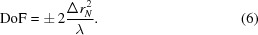
The achievable Δ_res_ depends only on Δ*r*_*N*_, whereas DoF depends both on Δ*r*_*N*_ and λ. The monochromaticity required to achieve equation (5) is λ/Δλ ≥ *N*, where *N* is the total number of zones. If this condition is not satisfied, both Δ_res_ and DoF become larger than equations (5) and (6) corresponding to the degree of monochromaticity. In this paper, hereafter, Δ_res_ is defined as a radius initially yielding effective null in the intensity distribution.

### 2.4. Relevant design parameters   

High-energy X-rays enable experiments to be conducted under atmospheric conditions, which is advantageous for the structural analysis of practical samples. Considering this, the operation X-ray energy of the IP-CZP is at the relatively high energy of 10 keV. The target Δ_res_ is set to be about 100 nm. Since the monochromaticity, λ/Δλ, of a silicon (111) double-crystal monochromator typically used in synchrotron radiation beamlines is of the order of several thousands, the effective total number of zones, 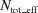 = 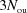, must be smaller than λ/Δλ. The focal length should not be too small in order to maintain a practical working distance.

In order to be consistent with our previous studies (Ozawa *et al.*, 1997 ▸), tantalum is chosen as the zone material. According to the data tables (Henke *et al.*, 1993 ▸; Center for X-ray Optics, http://henke.lbl.gov/optical_constants/tgrat2.html), the refractive index of tantalum at 10 keV is δ = 2.34356557 × 10^−5^ and β = 3.89601519 × 10^−6^. As an initial design step, the zone thickness of the iZP, *t*_in_, is chosen to be λ/2δ = 2.645 µm, yielding a phase shift of π, which means that the iZP works as an ordinary phase ZP when neglecting absorption.

Considering the above boundary conditions, *r*_1_in_ and *r*_1_ou_ are set to be 2.50 µm and 4.33 µm, respectively, which gives a focal length *f*_1_in_ of 50.41 mm at 10 keV. Δ*r*_*N*_in_ (= Δ*r*_fab_) is set to be 84 nm. Thus, *r*_*N*_in_ becomes 37.25 µm with *N*_in_ = 222. By modifying *N*_ou_ (equally ∊), η and ζ, the performance of Δ_res_ and DoF has been optimized.

## 3. Calculation of optical properties   

### 3.1. Diffraction integration   

**Figure 3:**
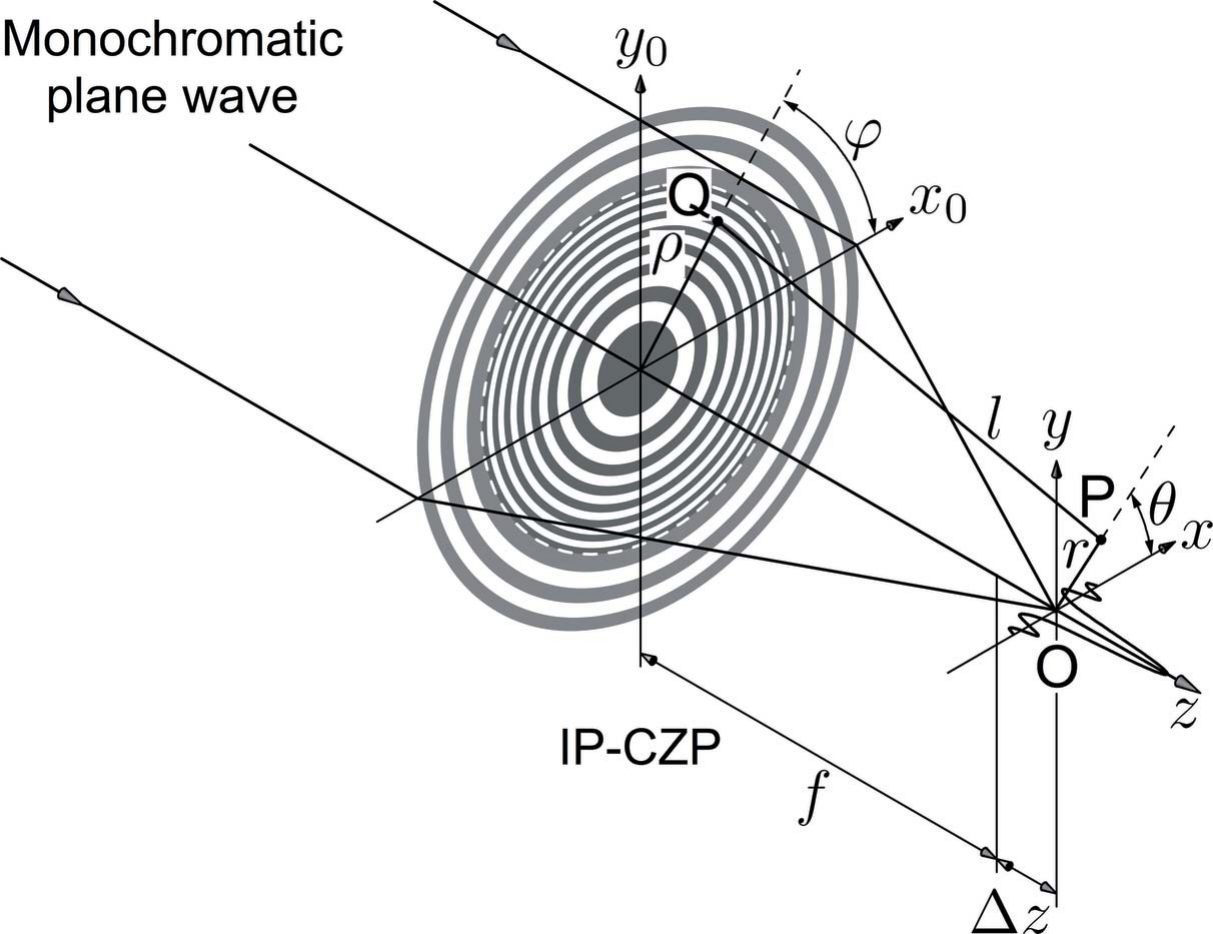
Optical system for diffraction integration with a monochromatic plane-wave illumination. (ρ, φ) and (*x*_0_, *y*_0_) are coordinates in the IP-CZP plane, and (*r*, θ) and (*x*, *y*) are those in the observation plane at a distance *z* (= *f* + Δ*z*) from the IP-CZP. *l* is the distance from a point *Q* in the IP-CZP plane to a point *P* in the observation plane.

The three-dimensional complex amplitude distribution neighboring the focal point, *A*(*r*, θ, *z*), has been calculated according to the diffraction integration with a monochromatic plane-wave illumination of wavelength λ propagating along the *z*-axis. The optical system is shown in Fig. 3 ▸. If those of the iZP and oZP are denoted independently as *A*_in_(*r*, θ, *z*) and *A*_ou_(*r*, θ, *z*), respectively, the diffraction integration is written as follows in cylindrical coordinates,
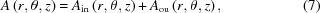


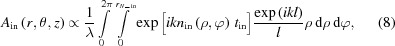


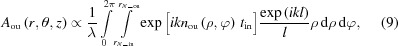


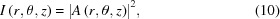


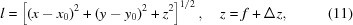


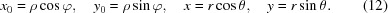
In the above, (ρ, φ) and (*x*_0_, *y*_0_) are polar and Cartesian coordinates in the IP-CZP plane, and (*r*, θ) and (*x*, *y*) are those in the observation plane at a distance *z* (= *f* + Δ*z*) from the IP-CZ. *l* is a distance from a point *Q* in the IP-CZP plane to a point *P* in the observation plane. 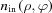 and 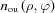 are the refractive index distributions of the iZP and oZP, respectively. *I*(*r*, θ, *z*) is the intensity. In the case when 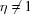 for the oZP, 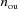 in the thickness direction between η*t*_in_ and *t*_in_, shown in Fig. 2(*b*) ▸, is replaced by that of the surrounding environment. This scalar wave simulation is valid for the evaluation of the proposed ICP-ZPs as discussed in Section S1 of the supporting information.

### 3.2. Two promising cases   

Diffraction integration has been performed to calculate the two-dimensional complex amplitude distribution (magnitude, phase, real and imaginary parts) neighboring the focal point. One dimension is the radial direction, *r*, and the other dimension is the optical axis direction, Δ*z*. The intensity is the sum of the squares of the real and imaginary parts.

Two characteristic promising cases were found: a pit-intensity focus with the deepest DoF (ZP-*A*) and a flat-intensity focus with deeper DoF (ZP-*B*) than a usual ZP. Table 1 ▸ shows parameters of the two promising cases. The iZPs are identical between the two. The diameter of the oZP of ZP-*A* is larger than that of ZP-*B* with ∊ of 0.782 and 0.836, respectively. The thickness of the oZP of ZP-*A* is larger than that of ZP-*B* with η of 1.00 and 0.85, respectively. The outermost zone width of the two oZPs, Δ*r*_*N*_ou_, is larger than twice that of the iZP, Δ*r*_*N*_in_, of 84 nm. The effective total number of zones, *N*_tot_eff_, is sufficiently smaller than several thousands, which means that the chromatic aberration is negligible in usual synchrotron radiation beamlines equipping standard silicon double-crystal monochromators.

**Figure 4:**
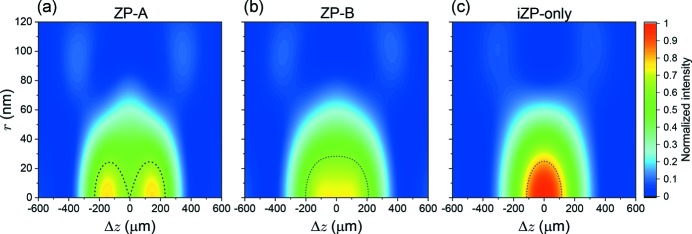
Calculated two-dimensional intensity distributions: (*a*) ZP-*A*, (*b*) ZP-*B* and (*c*) iZP-only. The intensity is a normalized value by the peak intensity of iZP-only. The black dashed lines denote contours of 80% intensity relative to self-peak intensity.

**Figure 5:**
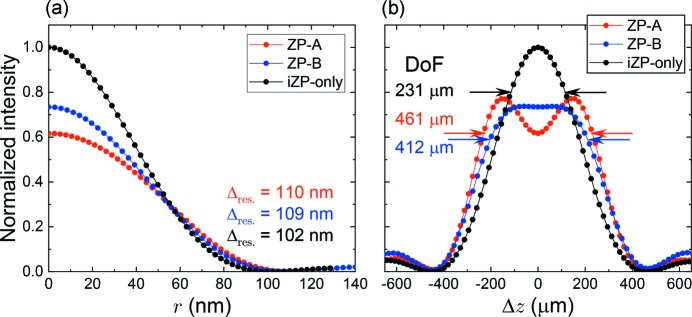
(*a*) Calculated point spread functions at Δ*z* = 0 and (*b*) intensity distributions along the optical axis, Δ*z*, at *r* = 0. The ordinate is a normalized value by the peak intensity of iZP-only. Δ_res_ of ZP-*A*, ZP-*B* and iZP-only are 110 nm, 109 nm and 102 nm, respectively. DoFs of ZP-*A*, ZP-*B* and iZP-only are 461 µm, 412 µm and 231 µm, respectively.

Fig. 4 ▸ shows the calculated two-dimensional intensity distribution, *I*(*r*, Δ*z*), of (*a*) ZP-*A*, (*b*) ZP-*B* and (*c*) iZP-only. The intensity is a normalized value by the peak intensity of iZP-only. The black dashed lines denote contours of 80% intensity relative to the self-peak intensity to make regions of each DoF clear. The ZP-*A* exhibits features of a pit-intensity focus and ZP-*B* exhibits features of a flat-intensity focus along the Δ*z* axis. Fig. 5 ▸ shows intensity distributions (*a*) in the radial-direction, *r*, at Δ*z* = 0 and (*b*) along the optical-axis, Δ*z*, at *r* = 0 of ZP-*A* and ZP-*B* accompanied by those of iZP-only. The former corresponds to the point spread functions (PSFs) and the latter determines DoF. The PSF of iZP-only [black in Fig. 5(*a*) ▸] is in accord with the theoretical values of 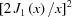, where 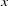 = 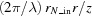 (Born & Wolf, 1986 ▸). The intensity distribution along the optical axis of iZP-only [black in Fig. 5(*b*) ▸] is also in accord with the theoretical values of 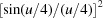, where 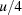 = 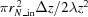 (Born & Wolf, 1986 ▸).

In this paper, Δ_res_ is defined as a radius initially yielding effective null in the intensity distribution. Each Δ_res_ is shown in Fig. 5(*a*) ▸. According to equation (4), the increase of DoF can be assessed. The calculation results relating to Δ_res_ and DoF are summarized in Table 2 ▸. The calculation results relating to intensity are summarized in Table 3 ▸. Δ_res_ is 110 nm and 109 nm, and DoF is 461 µm and 412 µm for ZP-*A* and ZP-*B*, respectively. Because Δ_res_ determined by Δ*r*_fab_ is 102 nm (= 1.22Δ*r*_fab_), the reduction in the spatial resolution is as small as 8% for both ZPs, from the point of view of fabrication. On the other hand, DoF can be 2.0 and 1.8 times deeper than that of iZP-only. The maximum intensity is reduced to 77% and 73% for ZP-*A* and ZP-*B*, respectively, in comparison with that of iZP-only. The integrated intensity, *I*_int_, within which the sum of intensities decreases by 20% from the self-peak intensity (inside the black dotted lines in Fig. 4 ▸), is reduced to 77% and 75% for ZP-*A* and ZP-*B*, respectively, in comparison with that of iZP-only. We found that a DoF of twice the depth could be expected with little reduction in the spatial resolution and with ∼1/4 intensity reduction.

It should be mentioned here that the focused intensity of ZP-*A* and ZP-*B* normalized by the total incident X-ray intensity, namely efficiency, is low due to both intentionally adapting the inverse phase of oZP and to larger diameters than that of iZP-only. The normalized integrated intensity by the area of the ZPs, which corresponds to efficiency, is also shown in Table 3 ▸. The efficiency of ZP-*A* and ZP-*B* is about half that of iZP-only. In synchrotron radiation beamlines, the incident beam size at the experimental station is usually larger than the size of practical ZPs. The larger incident beam size is also required to allow for the beam drift. Thus, the actual focused intensity may be more practically important than the efficiency.

### 3.3. Dependence on photon energy   

**Figure 6:**
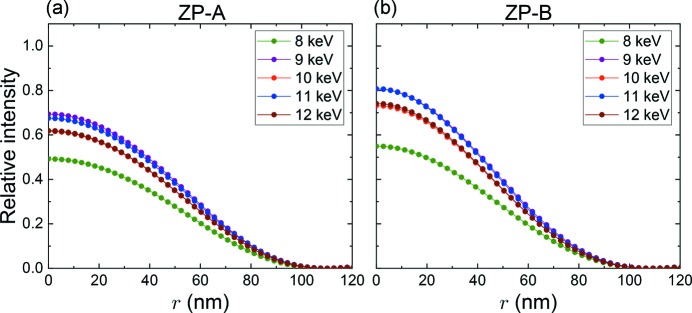
Calculated point spread functions at Δ*z* = 0 at several photon energies: (*a*) ZP-*A* and (*b*) ZP-*B*. The ordinate is a relative value to the peak intensity of iZP-only at the photon energy of 10 keV.

**Figure 7:**
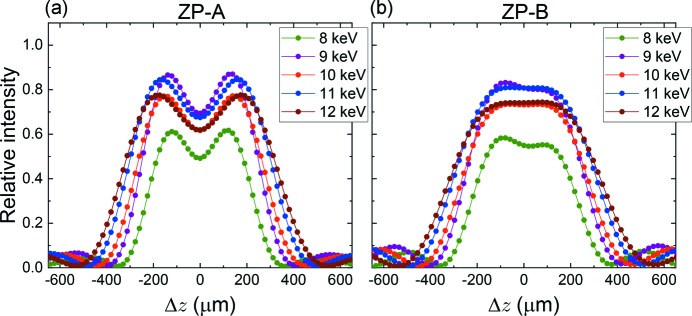
Calculated intensity distributions along the optical axis, Δ*z*, at *r* = 0 at several photon energies: (*a*) ZP-*A* and (*b*) ZP-*B*. The ordinate is a relative value to the peak intensity of iZP-only at the photon energy of 10 keV. The abscissa, Δ*z*, is a distance from a focus position of each photon energy.

**Figure 8:**
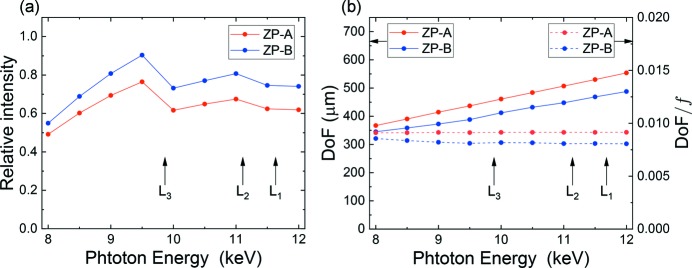
(*a*) Calculated intensity changes at Δ*z* = 0 and *r* = 0, and (*b*) DoF (left) and DoF/*f* (right) at several photon energies. The ordinate of (*a*) is a relative value to the peak intensity of iZP-only at the photon energy of 10 keV. In (*b*), the solid and dotted lines correspond to DoF and DoF/*f*, respectively. *L*-absorption edges of tantalum are indicated.

The focusing property dependence on photon energy has also been investigated. Fig. 6 ▸ shows PSFs of (*a*) ZP-*A* and (*b*) ZP-*B* for several photon energies from 8 to 12 keV, respectively. All PSFs self-normalized by the intensity at *r* = 0 have almost the same profile (not shown in the figure). Thus, the dependence of Δ_res_ on wavelength is negligible in the photon energy range. On the other hand, regarding intensity distributions along the optical axis, Δ*z*, at *r* = 0, the situation is somewhat different as shown in Fig. 7 ▸, where the abscissa, Δ*z*, is a distance from a focus position of each photon energy. ZP-*A* almost conserves the line symmetry about the line of Δ*z* = 0, while the line symmetry breaks for ZP-*B* in the lower photon energies. It is fortunate for ZP-*A* that the normalized intensity of the pits (Δ*z* = 0) by the self-maximum intensity is constantly 0.8, and thus the deepest DoF can be maintained in the photon energy range. These respective properties may not be simply understood, and further explanation is outside the scope of this paper. Fig. 8 ▸ shows (*a*) intensity changes at Δ*z* = 0 and *r* = 0, and (*b*) DoF and DoF/*f* changes. The diffraction efficiency of a phase zone plate depends on both the real part δ and the imaginary part β of the refractive index of the zone material (Kirz, 1974 ▸). The same theory can be applied to the present IP-CZP as a linear combination of complex amplitude of the iZP and oZP. The *L*-absorption edges of the zone material (tantalum) are indicated as a reference by vertical black arrows. The absorption edges have no influence on DoF. DoF increases linearly, while DoF/*f* is almost constant because *f* is proportional to the photon energy.

## 4. Future prospects   

Difficulties in the fabrication are not considered in this paper. Actually, the aspect ratio of the IP-CZP presented in this paper is 31.5, which is too high to realize by the presently popular fabrication technique of electron beam lithography. Solutions for fabricating high-aspect-ratio ZPs were demonstrated by stacking two identical zone plates (Snigireva *et al.*, 2007 ▸; Kagoshima *et al.*, 2011 ▸), double-sided zone plates (Mohacsi *et al.*, 2017 ▸) and zone plates of the highest aspect ratio fabricated by using metal-assisted chemical etching (Li *et al.*, 2017 ▸). Progress on these advanced fabrication technologies will make the proposed IP-CZPs available.

Another surpassing way to produce a blurred focal spot with an extended DoF has been studied (Li & Jacobsen, 2018 ▸). It uses multiple zone plate stacking with misalignment along the optical axis. The merit of producing blurred or extended DoF by multiple zone plate stacking is higher efficiency, *i.e.* zone plate stacking brings more energy to the focus. On the other hand, our IP-CZP has the great advantage that it is a single lens on a single substrate, and thus it can be easily handled and operated as a thin lens though the efficiency is low. Further, the focused intensity can be compensated by using brighter sources. Since Δ_res_ and DoF are naturally determined by the numerical aperture of an optical device itself, they are unable to be gained by the source intensity. Therefore, the IP-CZP will be practically valuable for thicker samples especially with future brighter sources.

## 5. Conclusions   

In order to defeat the diffraction limit restricting the relation between spatial resolution and depth of focus, an inverse-phase composite zone plate is proposed. The structure is a combination of an iZP functioning as a conventional phase zone plate and an oZP functioning with third-order diffraction with opposite phase to the iZP. The focusing properties of spatial resolution and DoF at a photon energy of 10 keV have been investigated by diffraction integration. Two characteristic promising cases were found of a pit-intensity focus with the deepest DoF and a flat-intensity focus with deeper DoF than usual ZPs. We found that a DoF of nearly twice the depth could be expected with little reduction in the spatial resolution, and that DoF and the spatial resolution were almost unchanged in the relatively wide photon energy range.

## 6. Related literature   

The following references, not cited in the main body of the paper, have been cited in the supporting information: Kang *et al.* (2005 ▸, 2006 ▸); Maser & Schmahl (1992 ▸); Schneider (1997 ▸); Schnopper *et al.* (1977 ▸).

## Supplementary Material

Section S1: validity of a scalar wave simulation; Section S2: .Properties of composite zone plates; Figure S1 and Tables S1 and S2.. DOI: 10.1107/S1600577518016703/pp5128sup1.pdf

## Figures and Tables

**Table 1:** Parameters of two promising IP-CZPs. ZP-*A* has a pit-intensity focus with the deepest DoF and ZP-*B* has a flat-intensity focus with deeper DoF than usual ZPs

ZP	*r*_1_in_ (µm)	*r*_*N*_in_ (µm)	*N*_in_	Δ*r*_*N*_in_ (nm)	*r*_1_ou_ (µm)	*r_N_*__ou_ (µm)	*N*_ou_	*N*_ou_eff_	Δ*r_N_*__ou_ (nm)	*N*_tot_eff_	∊	η	ζ
*A*	2.50	37.25	222	84	4.33	47.63	121	47	197	363	0.782	1.00	0
*B*						44.58	106	32	211	318	0.836	0.85	0

**Table 2:** Summary of the calculation results relating to Δ_res_ and DoF

ZP	Δ_res_ (nm)	FWHM (nm)	DoF (µm)	DoF Eq. (4) (µm)	DoF/DoF(iZP)	1.22Δ*r*_fab_ (nm)	Δ_res_/Δ_res_(iZP)
*A*	110	109	461	262	2.00	102	1.08
*B*	109	98	412	258	1.79	102	1.07
iZP-only	102	86	231	226	1	102	1

**Table 3:** Summary of the calculation results relating to intensity Values are relative values to those of iZP-only.

ZP	*I*_max_	*I*_int_	Normalized *I*_int_
*A*	0.77	0.77	0.47
*B*	0.73	0.75	0.52
iZP-only	1	1	1
