# Supplementary material for: Inverse-phase composite zone plate providing deeper focus than the normal diffraction-limited depth of X-ray microbeams
Source: J Synchrotron Radiat. 2019 Jan 1;26(Pt 1):52–8. doi: 10.1107/S1600577518016703 (PMC6337886; doi:10.1107/S1600577518016703)
Supplement: Supplementary file 1 [file s-26-00052-sup1.pdf]

JOURNAL OF SYNCHROTRON RADIATION

Volume 26 (2019)

Supporting information for article:

Inverse-phase composite zone plate providing deeper focus than the normal diffraction-limited depth of X-ray microbeams

Yasushi Kagoshima and Yuki Takayama

S1. Validity of a scalar wave simulation

The maximum aspect ratio of the IP-CZP presented in this paper is as high as 31.5. Validity using the diffraction integration (Eqs. (7) to (12), and Fig. 3) for such a high aspect ratio should be confirmed. According to previous works, when the zone plate thickness, t_{in}, is larger than $(2\Delta r_N)^2/\lambda$, the volume diffraction theory is required to calculate diffraction efficiency (Kang *et al.*, 2005, Kang *et al.*, 2006). If this condition is applied to the presenting case of tantalum zone plate (Δr_N = 84 nm and λ = 0.124 nm), $(2\Delta r_N)^2/\lambda$ = 228 μm, with which the aspect ratio is ~2700. Therefore, the volume diffraction theory is not necessarily needed for the presenting case. In order to reinforce the validity, calculations have been done on the diffraction efficiency both by the coupled wave theory (Maser and Schmahl, 1992, Schneider 1997, Koyama *et al.*, 2008) and the scalar diffraction theory (Center for X-Ray Optics, Schnopper *et al.*, 1977) for a tantalum zone plate. Figure S1 shows comparison between efficiency changes calculated by the coupled wave theory (red) and the scalar diffraction theory (blue) for the case of Δr = 84 nm. The bottom and top abscissas are tantalum zone thickness and the corresponding aspect ratio, respectively. The difference appears when the aspect ratio exceeds ~100. These calculation results reinforce the validity adopting the diffraction integration in this paper.

S2. Properties of composite zone plates

Composite ZPs (CZPs) with the same design parameters with the IP-CZPs are also of interest. A CZP-A and a CZP-B have the same parameters with ZP-A and ZP-B, respectively, except that $\zeta = \pi$. The calculation results relating to Δ_{res} and DoF are summarized in Table S1 and relating intensity are summarized in Table S2. Due to the constructive interference between iZP and oZP, both Δ_{res} and DoF become smaller than those of iZP-only. Similarly, I_{max} and I_{int} become about 1.5 and 1.3 times larger than those of iZP-only for CZP-A and CZP-B, respectively. On the other hand, the normalized intensity by the area of CZPs becomes smaller to be about 0.9. This is because of the nature of a CZP that the contribution of outer ZP to efficiency is much lower as being proportional to $1/9\times(r_{N\_ou}{}^2 - r_{N\_in}{}^2)$ than the contribution of inner ZP as being proportional to $r_{N\_in}{}^2$.

Table S1 Summary of the calculation results relating to Δ_{res} and DoF.

ZP	Δ_{res} (nm)	FWHM (nm)	DoF (µm)	DoF Eq. (4) (µm)	DoF/ DoF(iZP)	$1.22\Delta r_{fab}$ (nm)	Δ_{res}/ Δ_{res}(iZP)
CZP-*A*	93	76	162	188	0.70	102	0.91
CZP-*B*	95	79	185	197	0.80	102	0.93

Table S2 Summary of the calculation results relating to intensity. The values are relative values to those of iZP-only

ZP	I_{max}	I_{int}	Normalized I_{int}
CZP-*A*	1.47	1.48	0.90
CZP-*B*	1.32	1.32	0.92

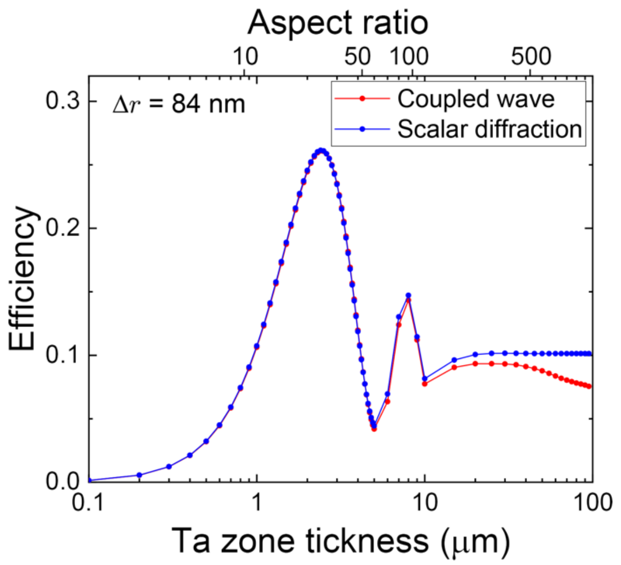

Figure S1 Comparison between efficiency changes calculated by the coupled wave theory (red) and the scalar diffraction theory (blue) for the case of Δr = 84 nm. The bottom and top abscissas are zone thickness and the corresponding aspect ratio, respectively.

References

Center for X-Ray Optics, Lawrence Berkeley National Laboratory. X-Ray Interactions With Matter, http://henke.lbl.gov/optical_constants/tgrat2.html.

Kang. H. C., Stephensona, G. B., Liu, C., Conley, R., Macrander, A. T., Maser, J., Bajt, S. & Chapman, H. N. (2005). *Appl. Phys. Lett.* **86**, 151109.

Kang, H. C., Maser, J., Stephenson, G. B., Liu, C., Conley, R., Macrander, A. T. & Vogt, S. (2006). *Phys. Rev. Lett.* **96**, 127401.

Koyama, T., Ichimaru, S., Tsuji, T., Takano, H., Kagoshima, Y., Ohchi, T. & Takenaka, H. (2008). *Appl. Phys. Express* **1**, 117003.

Maser, J. & Schmahl, G. (1992). *Opt. Commun.* **89**, 355.

Schneider, G. (1997). *Appl. Phys. Lett.* **71**, 2242.

Schnopper, H. W., Van Speybroeck, L. P., Delvaille, J. P., Epstein, A., Källne, E., Bachrach, R. Z., Dijkstra, J. & Lantward, L. (1977). *Applied Optics* **16**, 1088.
